# “SP-G”, a Putative New Surfactant Protein – Tissue Localization and 3D Structure

**DOI:** 10.1371/journal.pone.0047789

**Published:** 2012-10-18

**Authors:** Felix Rausch, Martin Schicht, Friedrich Paulsen, Ivan Ngueya, Lars Bräuer, Wolfgang Brandt

**Affiliations:** 1 Department of Bioorganic Chemistry, Leibniz Institute of Plant Biochemistry, Halle, Germany; 2 Institute of Anatomy, Department II, Friedrich Alexander University Erlangen-Nuremberg, Erlangen, Germany; Midwestern University, United States of America

## Abstract

Surfactant proteins (SP) are well known from human lung. These proteins assist the formation of a monolayer of surface-active phospholipids at the liquid-air interface of the alveolar lining, play a major role in lowering the surface tension of interfaces, and have functions in innate and adaptive immune defense. During recent years it became obvious that SPs are also part of other tissues and fluids such as tear fluid, gingiva, saliva, the nasolacrimal system, and kidney. Recently, a putative new surfactant protein (SFTA2 or SP-G) was identified, which has no sequence or structural identity to the already know surfactant proteins. In this work, computational chemistry and molecular-biological methods were combined to localize and characterize SP-G. With the help of a protein structure model, specific antibodies were obtained which allowed the detection of SP-G not only on mRNA but also on protein level. The localization of this protein in different human tissues, sequence based prediction tools for posttranslational modifications and molecular dynamic simulations reveal that SP-G has physicochemical properties similar to the already known surfactant proteins B and C. This includes also the possibility of interactions with lipid systems and with that, a potential surface-regulatory feature of SP-G. In conclusion, the results indicate SP-G as a new surfactant protein which represents an until now unknown surfactant protein class.

## Introduction

Surfactant proteins have been described in detail in relation with research on the lungs in which surface activity and immunological functions within both the specific and the non-specific immune defenses are ascribed to them [1], [2].

SP-A and SP-D are representatives of the C-type lectin family, in which other molecules with immunological properties can also be included. In accordance to the current understanding of the C-type lectin mechanism, the proteins bind to specific carbohydrates of bacteria, protozoans, fungi and viruses [3], [4]. This facilitates opsonization of and accelerated immune defense reactions to these microorganisms [5]–[7]. The presence of SP-A and SP-D with regard to their immunological function has been confirmed in various tissues, including human nasal mucosa, the digestive tract, tear ducts, salivary glands of the head and the gingiva [8]–[12].

In contrast to SP-A and SP-D, the small and extremely hydrophobic surfactant proteins SP-B and SP-C are essential components during formation of surfactant monolayers and the stabilization of air-fluid interfaces [1], [13], [14]. This extreme hydrophobicity of the surfactant proteins B and C is mostly obtained by posttranslational modifications. For example, the surfactant protein C is palmitoylated to increase its hydrophobic character [15]. Similar to SP-A and SP-D, the presence of SP-B and SP-C has already been demonstrated in a variety of tissues and humors, including tissues of the nasolacrimal apparatus and ocular surface, in tear fluid, in salivary glands, in the gingiva and in saliva [10], [11], [16].

While working with the four already known surfactant proteins, our attention was also attracted to another putative surfactant protein, which was identified by means of bioinformatic investigations and named surfactant protein G (SP-G) or surfactant-associated protein 2 (SFTA 2) [17]. The protein (SP-G) is encoded on the human chromosome 6, its primary theoretical translation product consist of 78 amino acid residues resulting in a molecular weight of approximately 8 kDa. This putative surfactant protein shows no sequential or structural similarities to surfactant proteins or other known proteins in general and therefore seems to represent a new group of proteins. Furthermore, there is no hard evidence or information neither on the organ or tissue distribution nor on the function of the protein. It is carrying an N-terminal signal peptide of 19 amino acid residues which is essential for protein secretion [18]. Therefore, there are probably other parts of the protein which show surface activity as well.

Since there are only a few already known facts about this protein available, choosing the right experimental work for further characterization can be very difficult. In such cases, computational methods like the protein structure modeling or molecular dynamics (MD) simulations can be very helpful. The generation of a three-dimensional (3D) model of the yet unknown protein structure can give hints about the solubility of the protein or possible interactions with solutes of its environment like lipids, sugars or other proteins. Furthermore, the model can show which parts of the protein are exposed to the solvent and in that way are most likely to carry posttranslational modifications. These are probably essential for the protein function [19], as already described for the known surfactant proteins [15], [20], [21]. The behavior of a protein in solution and possible interactions with other nearby solutes can be investigated by MD simulations. This method can calculate the time-dependent state of a system and in that way give a hint which dynamic processes a protein could perform. There are already MD simulations described in the literature, which showed the detailed interaction of SP-B with lipid monolayers [22], [23] and also demonstrated the crucial role of SP-B and SP-C for the preservation and formation of a stable lipid layer system on air-fluid surfaces [24], [25]. Similarly, MD simulations with SP-G could show if this protein can also interact with single lipids or lipid layers and with that, has functions comparable to the already know surfactant proteins.

The objective aim of our work was to combine both computational chemistry and experimental work to get further insights into the character and function of SP-G to show that this protein indeed has the potential to interact with lipid systems and is located in tissues where this functionality is very important (e.g. lung or ocular system). This will suggest SP-G in fact as a surfactant protein itself which represents an until now unknown surfactant protein class. Furthermore, the computational chemistry methods used in our work could assist during the development of potent antibodies to be able to investigate the tissue and organ specific distribution of the protein and therefore help to understand the function of this putative surfactant protein.

## Materials and Methods

### Tissues

The tissue samples were obtained from cadavers (5 male, 11 female, aged 33–76 years) donated to the Department of Anatomy and Cell Biology, Martin-Luther-University Halle-Wittenberg, Germany. These human tissue samples were obtained from body donors that donated their body to the Department of Anatomy and Cell Biology, Martin Luther University Halle-Wittenberg, Germany by testament. For this, each body donor singed a contract that he or she donates its body to the department mentioned above for research or teaching purpose. The contract was signed when the body donor was still alive. After death the respective body donor was transferred to the Department of Anatomy and Cell Biology, MLU Halle-Wittenberg and the tissue samples were obtained. This procedure is general practice in Germany and is in accordance with German and EU law. The study was approved by the Institutional Review Board of the Martin Luther University Halle-Wittenberg in accordance with the Declaration of Helsinki. The used samples were dissected from the cadavers within a time-frame of 5–20 h postmortem. Previous to dissection, the history of each cadaver was studied. Samples that were affected by acute infections, tumors, recent traumata or surgical operations were not used in this study. Furthermore, all samples with a post-mortal interval greater than 20 hours were omitted. After dissection, half of the specimens were fixed in 4% paraformaldehyde for later paraffin embedding. The other half of the specimens were used for molecular-biological investigations and thus immediately frozen at −80°C. For the experimental part, we used lung, testis, eye lid, heart, liver, kidney, parotid gland, lacrimal gland, cornea, conjunctiva, umbilical cord, trophoblast, stomach, spleen and nasal mucosa samples.

### Polymerase Chain Reaction (PCR)

For conventional PCR, we used conditions as previously described by us with the following primers: SP-G sense 5′- AGCGTGAGCAGGAAGGTTCT -3′, antisense 5′-GCGCCATGTAAGAGAGCTCT-3′ (ca. 250 bp) [11]. For verification and comparison, bacterial plasmids carrying the genes for the investigated protein were used as a reference (German Resource Centre for Genome Research GmbH; SP-G: IRAKp961J2287Q). PCR products were also confirmed by BigDye sequencing (Applied Biosystems, Foster City, CA). To estimate the amount of amplified PCR product, we performed a ß-actin PCR with specific primers (sense 5′- CAA GAG ATG GCC ACG GCT GCT-3′, antisense 5′- TCC TTC TGC ATC CTG TCG GCA-3′, 275 bp) for each investigated tissue. PCR products were also confirmed by BigDye sequencing (Applied Biosystems, Foster City, CA).

### Generation of Polyclonal Antibody

Anti-peptide antibody was generated against a specific region of the human SP-G sequence (YESSFLELLEKLCLLLHL). The peptide was synthesized by SeqLab (Göttingen, Germany). After coupling the peptide to keyhole limpet hemocyanin, it was used for the immunization of a rabbit. The polyclonal antibody was separated and enriched from the serum by Protein A-Sepharose. The polyclonal antibody was affinity purified by standard protocols. The specificity of the antibody was shown by Western blot analysis.

### Cloning of SP-G Using *E. coli*

For protein expression in *Escherichia coli*, the coding region without signal sequence was cloned into the pET100D vector containing a 6xHis tag using the pET Directional TOPO expression kit. Instructions were provided by the Champion™ pETDirectionalTOPO® Expression kit (Invitrogen Life Technologies, Carlsbad, CA). For the amplification of SP-G we used the following primers: sense 5′-CACCATGGGGTCTGGGCTG-3′ and antisense 5′-TCATGTGTTGCAGACAACAT-3′. The PCR products were ligated into the TOPO/TA vector and transformed into TOP100 *E. coli* cells (Invitrogen, Carlsbad, CA). Positive colonies containing the inserted gene in the proper orientation were identified using PCR.

### Western Blot Analysis

For Western blots, lung tissue (standardized ratio: 100 mg wet weight/400 µm buffer containing 1% SDS and 4% 2-mercaptoethanol) was extracted as previously described in detail by Bräuer [10]. The protein was measured with a protein assay based on the Bradford dye-binding procedure (BioRad, Hercules, CA). The total protein (30 µg) was then analyzed by Western blot. Proteins were resolved by reducing 15% SDS-polyacrylamide gel electrophoresis, electrophoretically transferred at room temperature for 1 h at 0.8 mA/cm^2^ onto 0.1 µm pore size nitrocellulose membranes and fixed with 0.2% glutaraldehyde in phosphate-buffered saline for 30 min. Bands were detected with primary antibody to SP-G (1∶250) and secondary antibody (anti-rabbit IgG, respectively, conjugated to horseradish peroxidase, 1∶5.000) using chemiluminescence (ECL- Plus; Amersham-Pharmacia, Uppsala, Sweden). Human lung was used as the control. The molecular weights of the detected protein bands were estimated using standard proteins (Prestained Protein Ladder, Fermentas, St. Leon-Rot, Germany) ranging from 10 to 170 kDa.

### Immunohistochemistry

For immunohistochemistry, tissue specimens from healthy tissues of body donors were embedded in paraffin, sectioned (6 µm) and dewaxed. Immunohistochemical staining was performed with the polyclonal antibody against SP-G. Antigen retrieval was performed by microwave pretreatment for 10 min and non-specific binding was inhibited by incubation with porcine normal serum (Dako) 1∶5 in Tris-buffered saline (TBS). Each primary antibody (1∶50–1∶100) was applied overnight at room temperature. The secondary antibodies (1∶300) were incubated at room temperature for at least 4 h. Visualization was achieved with diaminobenzidine (DAB) for at least 5 min. After counterstaining with hemalum, the sections were mounted in Aquatex (Boehringer, Mannheim, Germany). Two negative control sections were used in each case: one was incubated with the secondary antibody only, and the other one with the primary antibody only. The slides were examined with a Keyence Biorevo BZ9000 microscope.

### Protein Expression and Isolation

For the recombinant expression of SP-G, a system carrying an inducible T7 promoter and an N-terminal 6xHis tag was used. Overnight cultures of LB broth (5 ml) containing 0.02 mg/ml ampicillin (amp+) were used to inoculate the 500 ml LB/amp+ cultures. The cultures were induced with isopropyl-ß-D-thiogalactopyranoside (IPTG) in midlog phase (OD_600_ 0.6–0.8) and incubated at room temperature for 18–20 h. The cells were harvested by centrifugation and the supernatant was discarded. The cell pellet was resuspended in 20 mM Tris, 500 mM NaCl, and 5 mM imidazole with a pH of 7.9 (1/20 of the initial culture volume). The resuspended cells were placed on ice and lysed by ultrasonication. The soluble protein of the cell lysate was isolated by centrifugation at 13.000 rpm for 20 min at 4°C. The supernatant was use directly for Antibody testing.

### Protein Structure Model Creation

The first attempts to obtain a 3D-model of the protein structure were done by homology modeling with YASARA [26], [27]. Furthermore, the online servers I-TASSER [28], [29] and LOOPP [30], [31] were used, which follow the so called threading-approach. The final model for SP-G was obtained by the online *ab initio* folding server ROBETTA [32]. The resulting model was further processed by energy minimization and MD refinement [27] in YASARA to optimize the intramolecular interactions and stereochemistry of the structure model. Following this, PROCHECK [33] was performed to assess the stereochemical quality and PROSA II [34] was used to evaluate the quality of the entire protein fold or a partially misfolded structure. Prosa II contains knowledge based mean fields derived from statistical analysis of well resolved protein X-ray structures. Both validation programs can give clear hints if the structure model resembles a native-like fold. The final SP-G model was accepted by and deposited at the Protein Model DataBase PMDB [35] and received the PMDB id PM0078341 for free download.

To check the stability of the model, a 20 ns MD simulation was performed in YASARA [36], [37]. The MD was done in a water box with a physiological NaCl concentration of 0.9% and the YASARA2 force field [27].

### Prediction of Protein Modifications

Statistical sequence based prediction tools were used to analyze SP-G *in silico* for posttranslational modifications. Therefore, different programs were used which are linked over the ExPASy bioinformatics resource portal [38]. The protein sequence was scanned for acetylation, *N*-glycosylation, *O*-glycosylation with N-Acetyl-glucosamine (GlcNAc) or N-Acetylgalactosamine (GalNAc) and phosphorylation with NetAcet [39], NetNGlyc [40], NetOGlyc [41], YinOYang [42] and NetPhos [43], respectively. Moreover, CSS-Palm [44] was used to check if there is the possibility of palmitoyl chains bound to the two available cysteine residues. Predicted modifications were added manually to the protein structure model, followed by an energy minimization in YASARA. The resulting modified SP-G model was accepted by and deposited at the Protein Model DataBase PMDB [35] and received the PMDB id PM0078342 for free download. The stability of the modified 3D model was checked by a 20 ns MD simulation in YASARA similar to the calculation for the unmodified structure model (water box, 0.9% NaCl, YASARA2 force field).

### Molecular Dynamics Simulations

The system required for investigating possible interactions between the protein model and a lipid environment should be as close as possible to the native state. For this reason, dipalmitoylphosphatidylcholine (DPPC) was chosen for the MD simulations, since it is the major component of the lung surfactant [45]. To meet the present picture of the lung surfactant lipid system [46], the DPPC molecules were arranged as a monolayer patch with the polar head groups facing a liquid phase and the alkyl chains facing the air.

**Figure 1:**
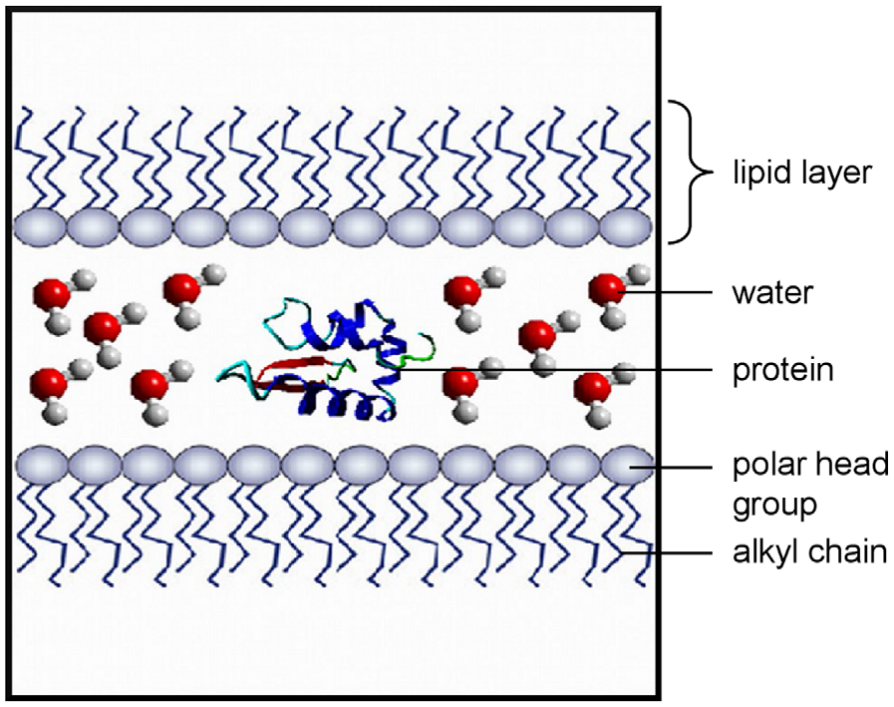
General simulation box setup. Two distinct DPPC lipid monolayers enclose a water phase (illustrated by single water molecules in ball and stick representation) with their polar head groups. The orientation of the protein (secondary structure only representation) centered in this water phase varies at the simulation start. On the alkyl chain side, the monolayers are separated by a vacuum phase because of the applied periodic boundary conditions.

The protein-lipid simulations were carried out with the GROMACS package version 4.5.4 [47], [48]. The united-atom G53a6 force field [49] was modified after Kukol [50] to produce reasonable data for a DPPC-lipid system. To allow the simulation of the modified protein models, the force field was extended by residues for phosphorylated serine, threonine and tyrosine, palmitoylated cysteine, serine or threonine residues which are *O*-glycosylated with GlcNAc or GalNAc and *N*-glycosylated asparagine. The residue for the *N*-glycosylation consists of a pentasaccaride core with two GlcNAc and three mannose moieties (- GlcNAc-GlcNAc-mannose-(mannose)_2_ ). Parameters for all these groups were taken from building blocks of the original G53a6 force field and in the case of the phosphorylated amino acids from the G43a1p force field [51]. The CELLmicrocosmos MembraneEditor 2.2 [52] was used to build the initial simulation system (Figure 1). It consists of two DPPC monolayers with 128 molecules each which are separated on the polar head group side by a water phase. On the side of the lipid alkyl chains the two layers are divided by a vacuum phase since periodic boundary conditions are applied in all three dimensions. For every simulation, one copy of the protein model was placed in different orientations in the water phase between the two lipid layers and was neutralized with Na^+^ or Cl^-^ ions, if necessary. This resulted in systems with a total size of approximately 60.000 atoms.

After a short equilibration period (500 ps), the simulations were carried out for 50 ns with the Nosé-Hoover thermostat [53], [54] at 323 K and the Parrinello-Rahman barostat [55], [56] with semi-isotropic coupling and a reference pressure of 1 bar. To maintain the simulation setup, the compressibility of the system in z direction was set to 0. The LINCS constraint algorithm [57], [58] was used to fix the stretching of bonds involving hydrogen atoms, allowing a time step of 2 fs. Electrostatic interactions were calculated with the particle mesh Ewald (PME) algorithm [59], [60] as implemented in GROMACS with a cutoff at 1.2 nm, the van der Waals potential was switched off between 1.2 and 1.3 nm. The neighbor list was updated every 10 steps and no dispersion correction was applied. The analysis of the simulations was done with the tools integrated in the GROMACS package. Visualization of the structures and trajectories was done with VMD [61] and YASARA.

## Results

### Modeling the Protein Structure of SP-G

**Figure 2:**
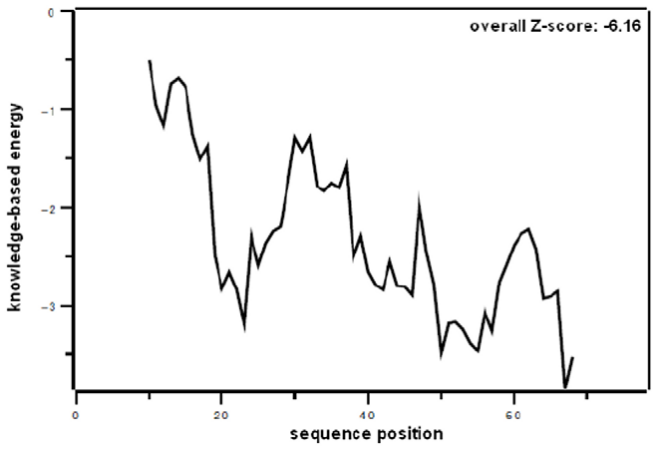
PROSA plot for the obtained SP-G structure model. The combined pair and surface potential is plotted versus the sequence position. The completely negative curve and the overall Z-score of −6.16 suggest a native-like fold.

The full length protein sequence (including the N-terminal 19 amino acid signal peptide) was used for the protein model generation because there are no data available which indicate in which form SP-G is present at its site of action. First attempts to obtain the 3D structure by homology modeling failed because there were no entries in the PDB with a sufficiently high sequence homology to SP-G. Also the threading method did not lead to satisfying results since the sequence of SP-G contains no conserved domains and the secondary structure prediction for a sequence with only 78 amino acids is very complicated. Therefore, the sequence was submitted to the online server Robetta. It applies *ab initio* folding to obtain a structural model in a very time consuming process. But for the short SP-G sequence, results were expected in reasonable time. Indeed, the obtained model showed a very promising quality and needed only minor optimizations. After energy minimization and MD refinement in YASARA, the PROCHECK evaluation shows a very good stereochemical quality of the protein model. From the 78 amino acids, 95.5% are in the most favored regions, the remaining 4.5% show dihedral angle values in the additional allowed regions of the Ramachandran plot. The evaluation with PROSA shows a very good model quality as well. The plot of the combined pair and surface potential (Figure 2) is clearly negative for all regions of the protein and the combined Z-score of -6.16 is close to the average value (−7.77) for proteins of this length. These validation results indicate a good native-like fold of the protein structure model.

**Figure 3:**
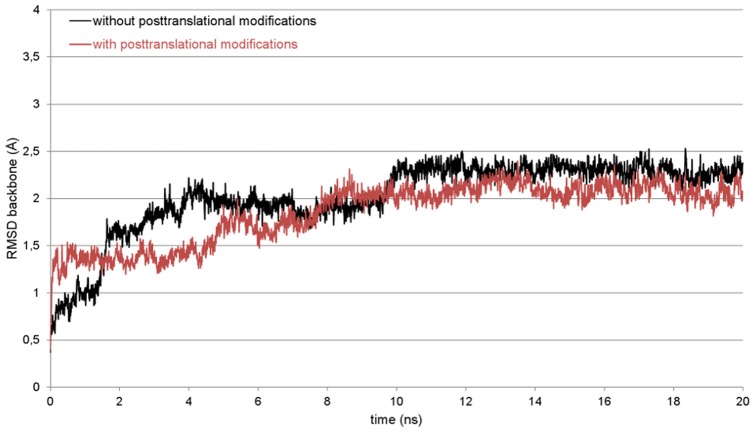
Root-mean-square deviation (RMSD) plot for the SP-G backbone atoms. The RMSD was calculated over the whole simulation process to check the stability of the unmodified (black) and posttranslationally modified (red) protein structure model. Rare and small plot fluctuations indicate a stable model.

Knowing that the overall quality of the protein model is appropriate for further studies, a 20 ns MD simulation in a water box was performed with YASARA which showed the model stability. There are no significant changes to the secondary structure visible and no hints for an unfolding of the protein structure can be observed. The results of the validation programs thereby are comparable to the aforementioned. The plot of the root-mean-square deviation (RMSD) over the simulation time as a measure for the distance between the starting and the resulting structure of the simulation also shows the stability of the 3D model (black plot, Figure 3). The RMSD reaches a plateau after about 10 ns over two clearly distinguishable phases. From this point, there are only very small fluctuations and the model can be considered as equilibrated.

**Figure 4:**
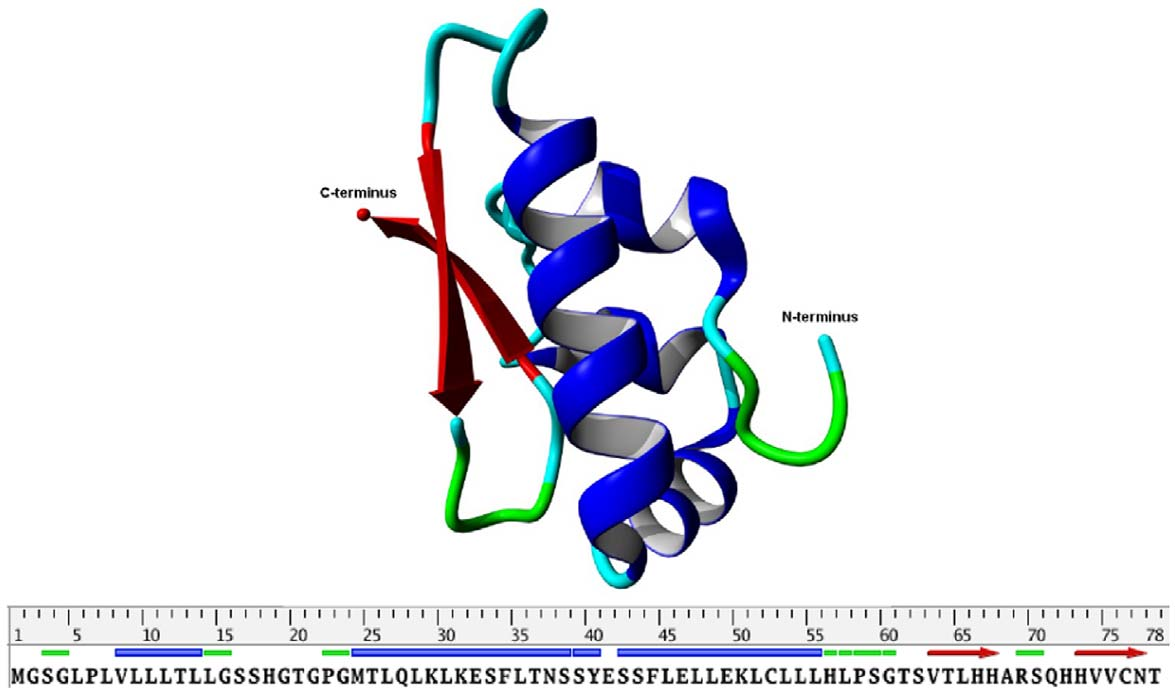
Structure presentation of the final protein model for SP-G (top). Only the protein backbone is shown in ribbon presentation. α-helices in blue, β-sheets in red, turns and random elements in green and cyan. The same color code is used on the sequence bar (bottom).

With this, we are the first who can present a three dimensional model of the SP-G structure. The model structure (Figure 4) is dominated by an α-helix of the amino acids 42–56 and an antiparallel β-sheet structure spanning the residues 63–68 and 72–78. The hydrophobic part of the N-terminal signal peptide is modeled as a short α-helix (8–13). This helix as well as the rest of the 19 N-terminal amino acids are loosely attached to the surface and cover the hydrophobic core of the protein. The fixation on the protein is not very strong so that it could fold out at any time to interact with or get embedded into a lipid system due to its hydrophobic character. The α-helix 42–56 also contains many hydrophobic residues (seven leucines and one phenylalanine). But in addition, it contains two glutamates and one lysine which could interact with the polar head groups of lipid moieties. Furthermore, the structure model shows that the two available cysteine residues are about 10 Å apart. This drastically reduces the possibility of an intramolecular disulfide bond. However, one of the cysteines (Cys76) is located on the surface of the protein and could be able to form an intermolecular disulfide bond, which would lead to a covalently connected protein dimer. Although there is no surface region predestined for interactions with another monomer, also a non-covalent oligomerization of SP-G cannot be excluded on the basis of the protein structure model.

### Protein Model with Posttranslational Modifications

Since it is known that posttranslational modifications are very important for the function of the already known surfactant proteins, SP-G was also analyzed for such modifications with different sequence based prediction tools. NetAcet shows no potential acetylation sites. NetNGlyc predicts an *N*-glycosylation on Asn37, whose occurrence is already noted in the SP-G UniProtKB entry. NetOGlyc predicts an *O*-glycosylation with GalNAc on the C-terminal Thr78. Overall, five *O*-glycosylations with GlcNAc as sugar moiety are predicted by YinOYang, whereat the probabilities for a modified Ser38, Ser39, Ser62 and Ser70 are quite moderate, but for a modification of Thr78 it is quite high. Given the results of NetPhos, the serines 17, 38 and 39 have a high phosphorylation potential, as well as Tyr40. Finally, the CSS-Palm server shows a potentially palmitoylated Cys76. On this point it is noticeable that there is only one prediction for the N-terminal signal peptide. This is a phosphorylation of Ser17, which is already very close to the signal peptide cleavage site. After all, the predicted posttranslational modifications were added manually to the structure model of SP-G, following two necessary conventions: 1. if there were several modifications predicted for the same position, only the modification with the highest probability was considered. 2. Only surface accessible amino acids were modified, since the addition of a bulky glycosylate moiety for example would have caused noticeably changes in the protein structure. The results of all predictions are summarized in Table 1, showing only the actually added posttranslational modifications. To examine if this extended model is stable, a MD simulation comparable to the one of the unmodified protein model was performed, i.e. with YASARA in a water box with 0.9% NaCl for 20 ns. The RMSD plot (red plot, Figure 3) clearly shows that the post-translationally modified model is very robust in this simulation system, reaching an equilibrium phase after 8 ns with only small RMSD fluctuations thereafter. As for the unmodified protein model, no significant secondary structure change or hints for an unfolding of the protein structure was observed. This indicates that the added modifications have no influence on the stability of the protein structure.

**Table 1:** Predicted posttranslational modifications and their sequence positions in the SP-G model.

sequenceposition	Ser17	Asn37	Tyr40	Ser62	Ser70	Cys76	Thr78
**modification**	PHOS	*N*-GLYC	PHOS	*O*-GLYC	*O*-GLYC	PALM	*O*-GLYC

Present modification types: phosphorylation (PHOS), palmitoylation (PALM), *O*-glycosylation with GlcNAc (*O*-GLYC), and *N-*glycosylation with a pentasaccaride core consisting of 2 GlcNAc and 3 mannose moieties (*N*-GLYC).

However, having a closer look on the now obtained model it is obvious that the posttranslational modifications can have a significant influence on the properties of the protein. The numerous predicted glycosylations for example could improve the solubility of the protein by masking hydrophobic spots on the protein surface and shielding the “hydrophobic core” of the protein from the polar environment in the case of an aqueous solvation. But they could also guide the protein to the surface of a lipid (mono-) layer via interactions with the polar lipid head groups. In the case of the simulation in a water box, the palmitoylation on Cys76 naturally tries to evade the water contact by entering the hydrophobic protein core. But in a lipid environment, this palmitoylation could point away from the protein surface, functioning as a membrane anchor similar to the signal peptide. This could stabilize the protein on the surface of a lipid layer or mediate its absorption into a membrane.

### Expression of Specific RNA Amplification Products

**Figure 5:**
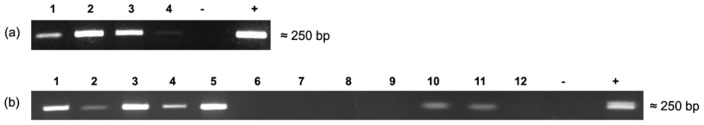
Expression of specific SP-G mRNA amplification products. The following samples were used: A) lacrimal gland [1], eyelids [2], conjunctiva [3], cornea [4]; B) lung [1], eyelid [2], heart [3], kidney [4], testis [5], stomach [6], spleen [7], nasal mucosa [8], salivary gland [9], umbilical cord [10], trophoblast [11], liver [12]; (−) negative control without template cDNA and (+) positive control – plasmid DNA carrying the ORF for the respective SP-G. All RT-PCR analyses show cDNA amplification for the corresponding SP in comparison with β -actin.

Tissue of the lacrimal gland, eyelids, conjunctiva and the cornea show presence of SP-G mRNA (Figure 5A). Tissue of lung, eyelids, heart, kidney, testis, umbilical cord and trophoblast show at least weak presence of SP-G mRNA (Figure 5B). In contrast, the tissues obtained from samples of stomach, spleen, nasal mucosa and salivary gland show no presence of SP-G mRNA. The ß-actin control PCR is positive for all samples (data not shown).

A special plasmid containing the full length gene served as a positive control for RT-PCR. The detected PCR bands are in accordance to the expected sequences within the gene bank data.

### Generation of a Specific SP-G Antibody

**Figure 6:**
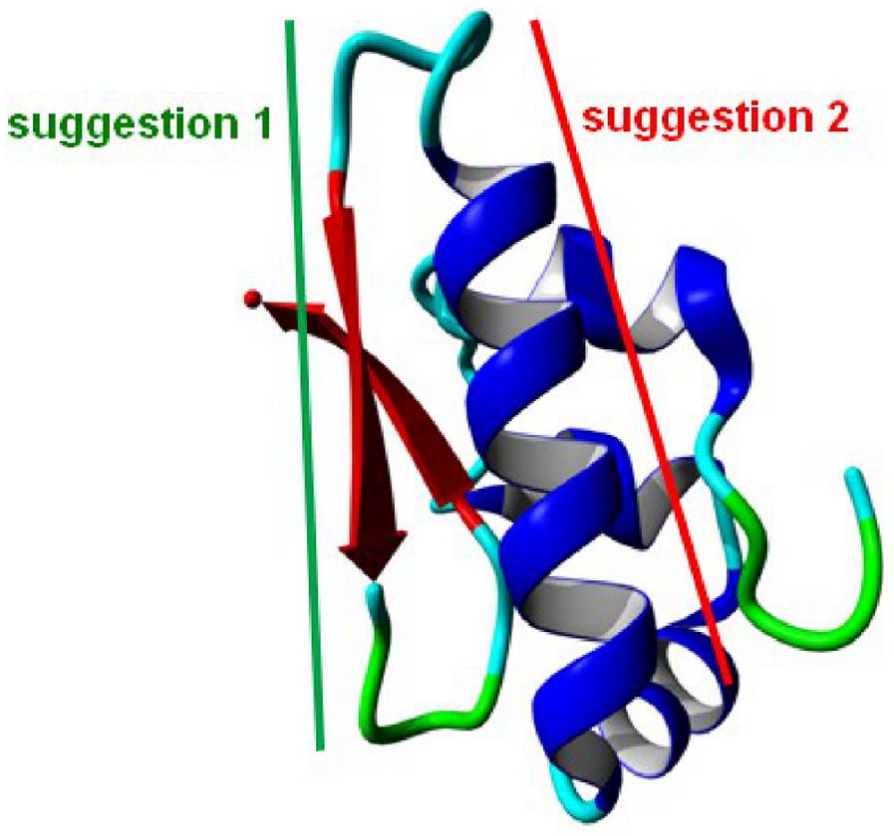
Two sections of SP-G suggested as potentially useful antigen regions. The sections are highlighted on the protein structure model. Suggestion 1 in green comprises a short β-strand, suggestion 2 in red covers an α-helix on the protein surface.

The protein structure model was used to identify a peptide sequence with the most promising specific protein-antibody interaction. For that, the peptide sequence should be as unique as possible in the proteome, on the surface of the protein and without any predicted posttranslational modification. Two areas of the protein could be identified which fulfill these criteria by looking at the 3D-model (Figure 6). The first suggestion comprises a beta-strand of the amino acids 60 to 70 (GTSVTLHHARS). This section is rather short and contains only one arginine and two histidines which could interact considerably with an antibody. The rest of this sequence part contains mainly hydrophobic amino acids. For this reason, this peptide sequence was not considered as a potent antigen. The second suggestion covers an α-helix ranging from position 40 to 57 (YESSFLELLEKLCLLLHL). It contains not only a lysine and a histidine, but also three negatively charged glutamates. These residues are very likely to form ionic interactions or hydrogen bonds with an antibody. Only the second peptide sequence was suggested for the antibody production.

**Figure 7:**
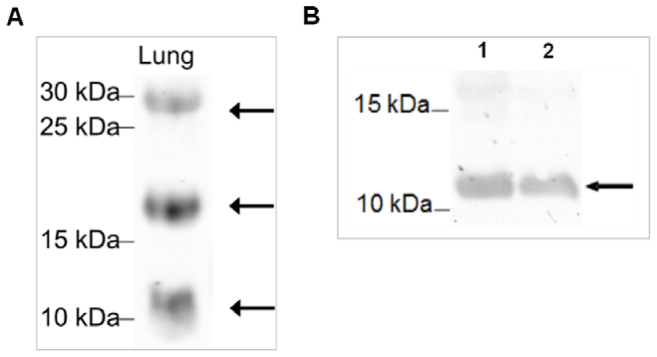
Antibody test by Western blot. Analyzed was A) lung tissue which is used as positive control for surfactant proteins; B) recombinantly synthesized SP-G protein (not purified) at 28°C (1) and 37°C (2), arrows indicate positive evidence of the surfactant protein G. The proteins extracted from the lung tissue and separated by 15% SDS-PAGE under reducing conditions show distinct bands for SP-G at the theoretically expected molecular weights of 11, 20 and 30 kDA [A]. In case of recombinantly expressed SP-G protein, the antibody detects a distinct band at 11 kDa [B].

The specificity of the resulting antibody was tested with protein from lung tissue (30 µg) and with the recombinantly synthesized SP-G protein (not purified, 30 µg) (Figure 7). The purified antibody shows distinct protein bands in lung for SP-G at 11 kDa, 20 kDa and 30 kDa and a distinct protein band for recombinantly synthesized SP-G at about 12 kDa. We used lung tissue as specific positive control for surfactant proteins.

### Detection and Distribution of SP-G in Human Tissue Samples by Means of Immunohistochemistry

**Figure 8:**
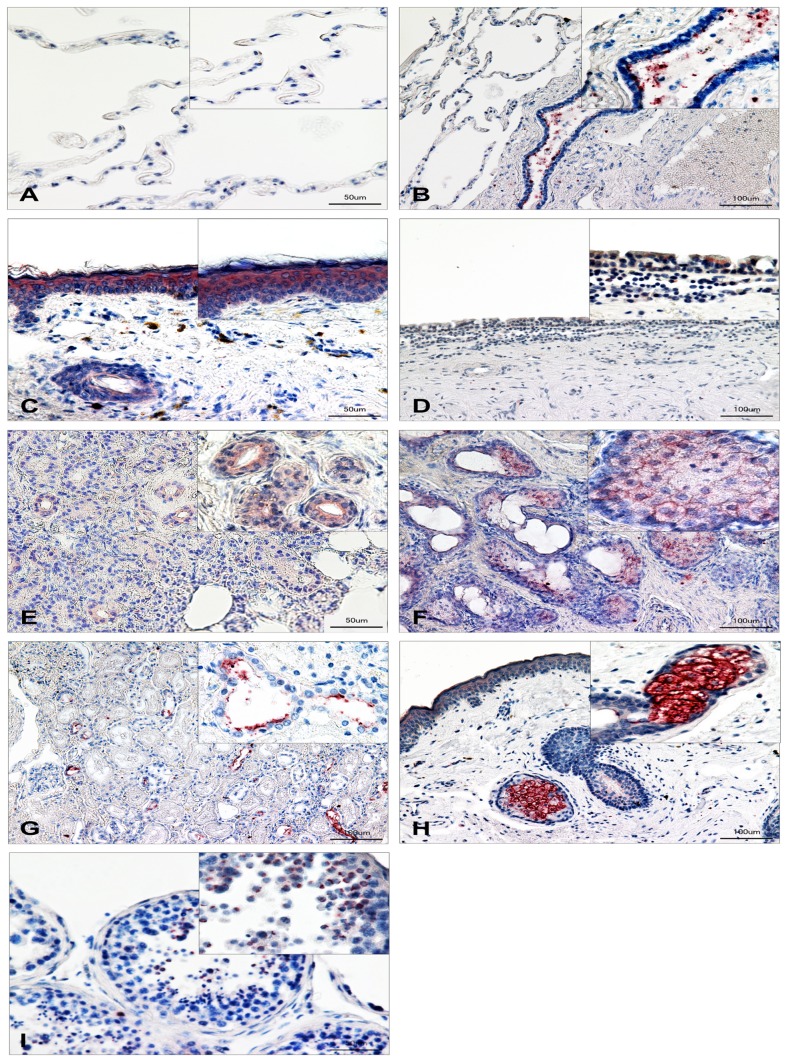
Immunohistochemical detection of SP-G in human tissue samples. Sections from lung [A-B], eyelid [C], conjunctiva [D], meibomian glands [E], lacrimal gland [F], kidney [G], sebaceous gland [H], and testis [I] were analyzed. Red stained areas within the tissues indicate SP-G expression. Insets in the figures show magnifications for the respective tissue. Control sections (secondary antibody only) were negative (unstained) for each tissue. Scale bars in [A, C, E, I] 50 µm, in [B, D, F, G, H] 100 µm.

All investigated tissue samples show antibody reactivity against SP-G (Figure 8A, B, C, D, E). Paraffin-embedded 6-µm sections from lung, eyelid, conjunctiva, meibomian glands, lacrimal gland, kidney, sebaceous gland and testis were analyzed. Control sections (secondary antibody only) were negative (unstained) for each tissue. The insets of the figures show magnifications for the respective tissue. For all tissues, red staining reveals positive antibody reaction for surfactant protein G. **Lung:** In the lung, SP-G could be detected as a superficial layer of the epithelium of the bronchioles. The alveolar membrane, especially the alveolar cells (I & II) are negative (Figure 8A, B).

#### Epithelium of the eyelid

SP-G was detected in the epithelium of the eyelid nearly equally distributed within the layers of the epidemis (Figure 8C). The subcutaneous tissue shows no or only weak reactivity against the antibody. **Conjunctiva:** The multilayer epithelium of the conjunctiva shows only weak reactivity (Figure 8D). In goblet cells of the conjunctiva, no presence of SP-G could be demonstrated. **Meibomian glands:** The meibomian glands in the investigated eyelids show reactivity against SP-G antibody, especially intracytoplasmatically within acinar cells and the excretory duct system (Figure 8E).

#### Lacrimal gland

Antibody reactivity displays the presence of SP-G intracytoplasmatically within acinar cells and in cells near the lumen of the intralobular duct system (Figure 8F).

#### Kidney

In the parenchyma of human kidneys we were able to detect antibody reactivity mostly in the cells of the ascending as well as the descending limb. Nephrons and glomerular cells show no presence of SP-G (Figure 8G).

#### Sebaceous glands

Sebocytes surrounding hair follicles show intense intracytoplasmic antibody reactivity against SP-G, whereas the follicles itself do not react with the antibody (Figure 8H).

#### Testis

Within seminiferous tubules, SP-G was only detected in spermatocytes and on the surface of the sperms (Figure 8I). Sustentacular cells of Sertoli, interstitial cells of Leydig as well as the epididymal tissue show no immunohistochemical evidence of SP-G (Figure 08).

### Simulation of the Protein Models in a Lipid Environment

**Figure 9:**
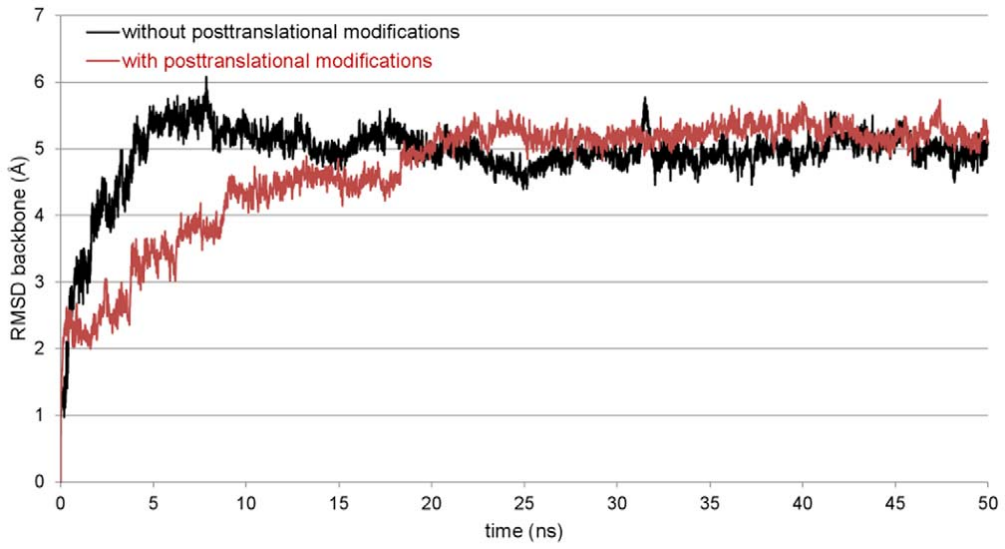
RMSD plots of SP-G models during 50 ns MD simulation. A representative simulation for the unmodified and posttranslationally modified SP-G model is shown in black and red, respectively. Both structure model variants are stable with respect to the RMSD plots of the backbone atoms.

**Figure 10:**
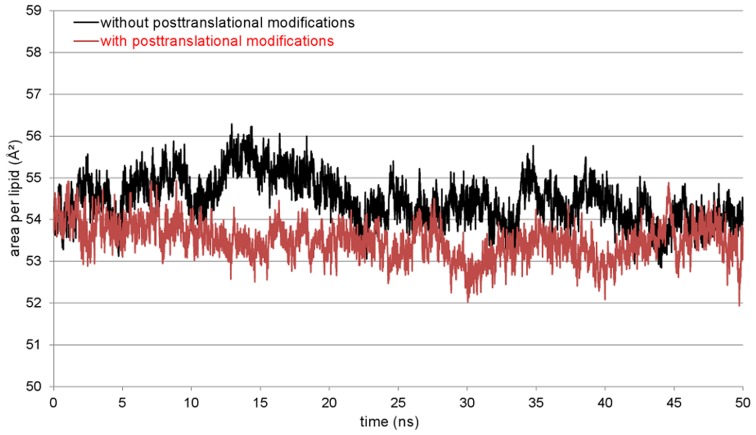
Plot of the occupied area per DPPC molecule. The calculation was done for the x-y-plane of the monolayer and the whole simulation time. The simulation for the unmodified (black) as well as for the posttranslationally modified (red) SP-G model shows a stable occupied area.

**Figure 11:**
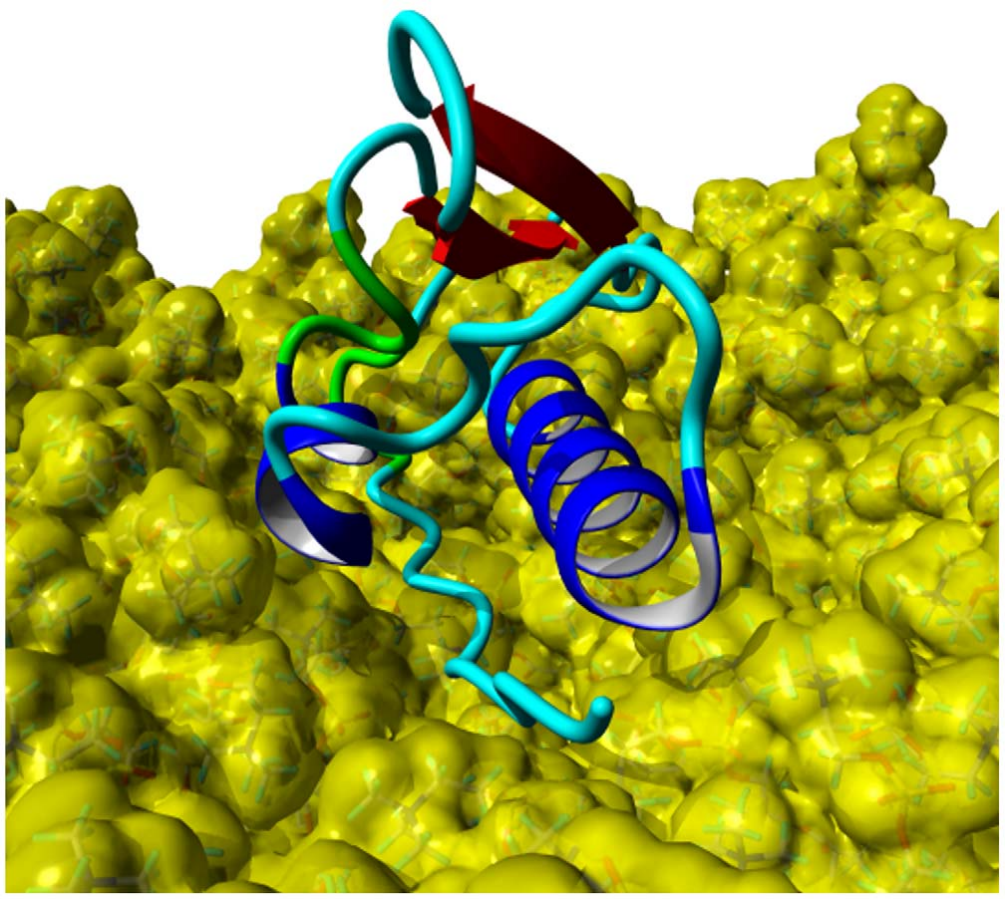
SP-G model and DPPC monolayer after a 50 ns MD simulation. The protein is shown in ribbon presentation (α-helices: blue, β-sheets: red, turns: green, coil: cyan) and the lipids with a yellow van der Waals surface. The N-terminal signal peptide and the α-helix 42–56 are interacting with the lipid surface, stabilizing the protein structure.

Four MD simulations of 50 ns length each were performed for the protein model without posttranslational modifications, starting from different orientations of the protein between the monolayers. The protein model moved in direction to one of the monolayers in all simulations, mostly interacting with the N-terminal signal peptide first. Thereby, the signal peptide is aligned parallel to the lipid surface after reaching the polar lipid head groups. It has to be noted that the α-helical conformation of this part is lost during this process. This position of the protein also allows the interaction of the α-helix 42–56 with the monolayer in a parallel orientation. This agglomeration process took between 2 and 10 ns of simulation time. The interactions between protein and lipids are stabilized during the remaining simulation time, leading to a very stable complex of protein and lipids. This stability is visible in both the protein backbone RMSD (black plot, Figure 9) and area per lipid plot (black plot, Figure 10). During this steady phase after about 15 ns, the hydrophobic part of the signal peptide is penetrating deeper into the lipid layer (Figure 11). There, the clearly visible interaction contact between the protein model and the lipid layer after 50 ns of MD simulation is shown.

**Figure 12:**
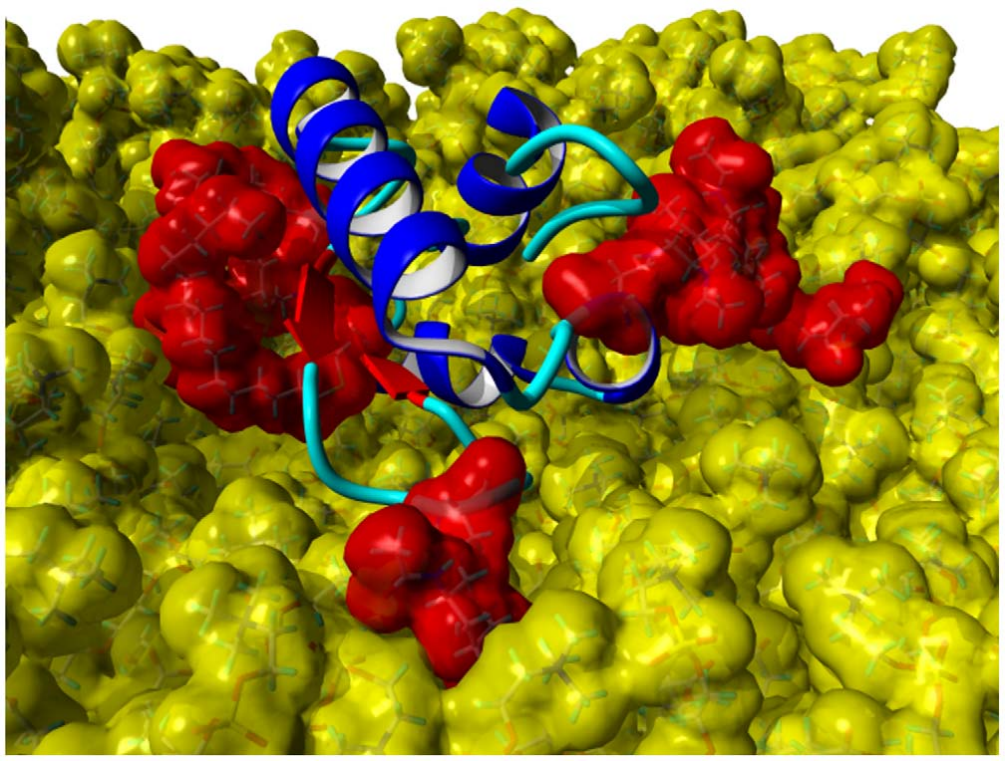
Posttranslationally modified SP-G model with DPPC monolayer after 50 ns MD simulation. The lipids are shown with a yellow, the posttranslational modifications at position 37, 62, 70, 76, and 78 are shown with a red van der Waals surface to illustrate the tight interactions. Furthermore, the protein backbone is shown with the ribbon presentation (α-helices: blue; β-sheets: red, coil: cyan).

Simulations with the same conditions were started for the SP-G model carrying posttranslational modifications as well. But in this case, a totally different interaction site between protein and lipids was obtained. First calculations indicated that the attached palmitoylation could interact with the lipid surface. For this reason, a simulation was started where the palmitoyl chain was already interacting with the monolayer at the beginning. After 50 ns MD simulation, the resulting protein-monolayer complex was analyzed (Figure 12). The α-helix 42–56 is on the surface of the complex, interacting with the solvent and not with the lipid surface as for the unmodified model. Instead, the palmitoylation on Cys76 and the protein part ranging from position 15 to 30 are interacting considerably with the lipids, the latter even immersing deep into the monolayer. The α-helical character of the signal peptide is maintained over the whole simulation. It is covering the hydrophobic protein core and is nicely stabilized in this conformation. The position of the whole protein on the lipid surface is fixed on three points by the attached glycosylations, which can interact significantly with the lipid head groups and act like anchors. This is also the reason why the protein RMSD plot for this simulation (red plot, Figure 9) is very stable. However, this strong interaction and the deep immersion of the protein into the lipid system affect the monolayer behavior. As for example, the area per lipid (red plot, Figure 10) is still essentially stable, but on average significantly lower than for the protein model without modifications.

In summary, it can be noted that with the means of the performed MD simulations, two potential poses for a possible interaction of SP-G with a lipid system could be identified. One scenario, where the interaction between protein and lipids is driven by the N-terminal signal peptide and the slightly hydrophobic α-helix 42–56 and another scenario, where the attached palmitoylation determines the interaction site and the resulting protein-lipids complex is stabilized by glycosylations. Both situations are until now valuable suggestions, but will have to be further investigated by experimental studies. However, these results suggest that SP-G has a great potential to interact with or have an influence on lipid systems, as it is already described for known surfactant proteins. Furthermore, the simulations showed that the posttranslational modification pattern of the protein can have a huge influence on the possible interactions of the protein and with that, is of great importance for the protein function.

## Discussion

By means of genome analysis, Zhang was able to identify the putative surfactant protein G (SP-G or SFTA2) [17] for the first time. This small protein is located and encoded on human chromosome 6p21.33. Its primary translation product comprises 78 amino acids leading to a premature peptide with a predicted molecular weight of approx. 8 kDa. Our results reveal that SP-G seems to be an amphiphilic protein which is able to switch between a hydrophilic and a more hydrophobic state. Furthermore, it has similar physicochemical properties like the surfactant proteins B or C, but without sequential and structural identities to the known surfactant proteins.

The objective of this study was the detection and characterization of SP-G within different human tissues (e.g. ocular surface, lung, kidney and testis). Because of lacking antibodies and structural information of the protein, we combined computational chemistry (protein modeling and MD simulation) and molecular-biological methods and were for the first time able to present a 3D protein structure model of the putative surfactant protein G. The obtained 3D-model indicated that SP-G seems to have hydrophobic properties and is most likely posttranslational modified with phosphorylations, glycosylations and palmitoylations, similar to the known surfactant proteins SP-B and SP-C [62], [63]. Our RT-PCR results show presence of SP-G mRNA within tissues of the ocular system and in the lung, kidney, heart, testis, umbilical cord, and trophoblast. Furthermore, with the 3D structural model in our hands, a specific peptide sequence of the protein was identified (YESSFLELLEKLCLLLHL) that showed promising antibody binding features because it is located on the surface of the protein and lacks any predicted posttranslational modifications.

The suggested sequence (YESSFLELLEKLCLLLHL) was chosen as a target peptide for the development of antibodies. For testing the antibodies, we performed Western blot analysis using human lung tissue, because all known surfactant proteins were identified and characterized also in the lung [1], [2], [13], [64], [65]. Furthermore, a very recent study of Mittal *et al.* also demonstrated the presence of SP-G within lung tissue [66]. The corresponding Western blot analysis show specific bands at 11 kDa, 20 kDa and 30 kDa. Considering that the protein might be posttranslational modified due to glycosylation, phosphorylation and palmitoylation, the distinct protein band at 11 kDa seems to represent the mature protein. Furthermore, the calculated molecular weight of the modified protein model confirms this observed value.

From literature, it is known that the surface active properties of SP-B and SP-C result from their intense posttranslational modifications [67]. To see if this is also the case for SP-G, sequence-based prediction tools were used to scan the SP-G sequence for modifications. The results suggest that SP-G is indeed highly posttranslationally modified as well. Furthermore, the performed MD simulations showed that the function of SP-G could be influenced by these modifications significantly.

For SP-B and SP-C, the modifications and additional intermolecular disulfide bonds allow the oligomerization of the proteins resulting in different molecular weights and different functions [62], [68]–[70]. The molecular weights for SP-B vary from 8 kDa and 25 kDa in the lung up to 35 kDa in tissues of the ocular system [16]. Also SP-C shows strong posttranslational modifications resulting in molecular weights differing from 7 kDa [71], 21 kDa [9] up to 26 kDa [72].

To exclude the possibility of non-specific antibody reactivity (cross reactions), we pre-incubated the antibody using the SP-G-peptide to block all antibody-binding sites. The corresponding Western blot analysis showed no protein bands (data not shown).

Within lung tissue, we could demonstrate that SP-G is distributed as a superficial layer of the epithelium of the bronchioles, which additionally indicated surface activity of the protein.

The existence of SFTA2 in type 2 pneumocytes was shown by the group of Mittal [66]. We cannot confirm this result but it is possible that cryo-slides are more suitable.

Bernhard *et al.* and Khoor *et al.* demonstrated that SP-B and SP-C are present in small concentrations in the secretions of the upper respiratory tract [73], [74]. In this context, other proteins originally not belonging to the group of surfactant proteins but also assigned to surface regulatory functions have been identified within secretions of the upper respiratory tract. One of these proteins is the PLUNC protein (Palate Lung Nasal Clone), which is a strong hydrophobic secretory product of the bronchial epithelium, of the lung and the upper respiratory tract [75], [76], [77]. At least theoretically, PLUNC shows a posttranslational modification comparable to SP-G, assuming similar physicochemical properties and features.

Furthermore, similar to the other known surfactant proteins, SP-G could be detected within epithelia of the ocular surface, amongst them conjunctiva, meibomian glands, accessory lacrimal glands, sebaceous glands and epidermis of the eyelids [9], [16]. The detection of SP-G within the lipid-containing sebaceous glands and within the meibomian glands enhances the theoretically evidenced palmitoylation and the resulting hydrophobic properties of SP-G. In contrast, the presence of SP-G within the acini of the serous lacrimal gland may reveal a hydrophilic character of the protein. This would be in accordance to the results of Mittal *et al.*, who postulated that SP-G (SFTA2) seems to have hydrophilic properties [66]. In this context, we suppose that, similar to SP-B and SP-C, also SP-G is an amphiphilic protein, showing both hydrophobic as well as hydrophilic properties depending on the posttranslational modifications. This behavior could also be managed by a dynamic process, as could be observed during the MD simulations of the posttranslationally modified protein model. The palmitoylation for example could be embedded into the hydrophobic core of the protein, changing the protein surface properties significantly. Also the hydrophobic N-terminal signal peptide is an important factor, since it can protrude from the protein surface or can be tightly bound to it. In that way, not only the shape of the protein is altered, but also the position of hydrophobic spots on the protein surface.

Furthermore, the MD simulations reveal the formation of domains within the SP-G protein that have the capability to interact with lipid phases. These domains and the palmitoylation of the protein also support the hypothesis that SP-G may be integrated into and anchored within a lipid phase, as can be found within actual accepted models of the tear film [78], [79]. Our calculations showed that SP-G is able to reside in vicinity of lipid systems and may also interact with them.

Inside the lacrimal gland, SP-G was demonstrated in the superficial cells of the excretory duct system, assuming that the protein might have also rheological properties and promotes the flow of tears. Similar effects of other surfactant proteins have already been demonstrated within the nasolacrimal duct [16], the salivary gland system [10] and Eustachian tube [80], [81].

SP-G could also be detected in the parenchyma of the kidney, mostly within the ascending as well as descending limb. Putative rheological functions of SP-G with respect to the tubular system of the kidney can only be supposed. So far, only the immunological surfactant proteins A and D have been detected in the kidney [81]. In this context, SP-G could be a new surfactant protein, performing rheological functions within the kidneys.

SP-G was also detected in human testes, but in this special case within spermatozoa. The function of the protein within testes and spermatozoa is quite speculative. Recent findings of Annalaura *et al.* demonstrated the presence of SP-B and SP-C in spermatozoa of whales [82]. Considering these findings and the proposed putative rheological properties, SP-G could be a new surfactant protein within testes that could assist during transport and facilitation of spermatozoa through the testicular duct system.

In summary, we have identified SP-G (SFTA2) as a novel secretory surfactant protein expressed in different tissues (lung, eyelid, kidney, and testis) on mRNA and protein level. The physicochemical similarity to the surfactant proteins B and C and the performed protein modeling studies and MD simulations indicate surface-regulatory properties of SP-G. A role of the protein in inflammation and immunological defense is speculative, because immune regulatory domains could not be identified, neither with the applied computational methods nor with the performed molecular-biological methods.

## References

[1] Yu SH, Possmayer F (1990) Role of bovine pulmonary surfactant-associated proteins in the surface-active property of phospholipid mixtures. Biochim Biophys Acta 1046: 233–241. 2223863 10.1016/0005-2760(90)90236-q

[2] Crouch E, Wright JR (2001) Surfactant proteins a and d and pulmonary host defense. Annu Rev Physiol 63: 521–554. 11181966 10.1146/annurev.physiol.63.1.521

[3] Hartshorn KL, Crouch E, White MR, Colamussi ML, Kakkanatt A, et al (1998) Pulmonary surfactant proteins A and D enhance neutrophil uptake of bacteria. Am J Physiol 274: L958–969. 9609735 10.1152/ajplung.1998.274.6.L958

[4] Ferguson JS, Voelker DR, McCormack FX, Schlesinger LS (1999) Surfactant Protein D Binds to Mycobacterium tuberculosis Bacilli and Lipoarabinomannan via Carbohydrate-Lectin Interactions Resulting in Reduced Phagocytosis of the Bacteria by Macrophages1. J Immunol 163: 312–321. 10384130

[5] van de Wetering JK, van Golde LMG, Batenburg JJ (2004) Collectins: players of the innate immune system. Eur J Biochem 271: 1229–1249. 15030473 10.1111/j.1432-1033.2004.04040.x

[6] Wright JR (2005) Immunoregulatory functions of surfactant proteins. Nat Rev Immunol 5: 58–68. 15630429 10.1038/nri1528

[7] Kishore U, Greenhough TJ, Waters P, Shrive AK, Ghai R, et al (2006) Surfactant proteins SP-A and SP-D: structure, function and receptors. Mol Immunol 43: 1293–1315. 16213021 10.1016/j.molimm.2005.08.004

[8] Bourbon JR, Chailley-Heu B (2001) Surfactant proteins in the digestive tract, mesentery, and other organs: evolutionary significance. Comp Biochem Physiol A Mol Integr Physiol 129: 151–161. 11369540 10.1016/s1095-6433(01)00312-9

[9] Bräuer L, Kindler C, Jäger K, Sel S, Nölle B, et al (2007) Detection of Surfactant Proteins A and D in Human Tear Fluid and the Human Lacrimal System. Invest Ophthalmol Vis Sci 48: 3945–3953. 17724171 10.1167/iovs.07-0201

[10] Bräuer L, Möschter S, Beileke S, Jäger K, Garreis F, et al (2009) Human parotid and submandibular glands express and secrete surfactant proteins A, B, C and D. Histochem Cell Biol. 132: 331–338. 10.1007/s00418-009-0609-x 19484255

[11] Bräuer L, Schicht M, Stengl C, Heinemann F, Götz W, et al (2012) Detection of surfactant proteins A, B, C, and D in human gingiva and saliva. Biomed Tech (Berl) 57: 59–64. 22718593 10.1515/bmt-2011-0031

[12] Kim JK, Kim S-S, Rha KW, Kim C-H, Cho JH, et al (2007) Expression and localization of surfactant proteins in human nasal epithelium. Am J Physiol Lung Cell Mol Physiol 292: L879–884. 17209137 10.1152/ajplung.00156.2006

[13] Notter RH, Shapiro DL, Ohning B, Whitsett JA (1987) Biophysical activity of synthetic phospholipids combined with purified lung surfactant 6000 dalton apoprotein. Chem Phys Lipids 44: 1–17. 3607971 10.1016/0009-3084(87)90002-8

[14] Curstedt T, Johansson J, Barros-Söderling J, Robertson B, Nilsson G, et al (1988) Low-molecular-mass surfactant protein type 1. Eur J Biochem 172: 521–525. 3350011 10.1111/j.1432-1033.1988.tb13918.x

[15] Beers MF, Mulugeta S (2005) Surfactant Protein C Biosynthesis and Its Emerging Role in Conformational Lung Disease. Annu Rev Physiol 67: 663–696. 15709974 10.1146/annurev.physiol.67.040403.101937

[16] Bräuer L, Johl M, Börgermann J, Pleyer U, Tsokos M, et al (2007) Detection and Localization of the Hydrophobic Surfactant Proteins B and C in Human Tear Fluid and the Human Lacrimal System. Curr Eye Res 32: 931–938. 18027169 10.1080/02713680701694369

[17] Zhang Z, Henzel WJ (2004) Signal peptide prediction based on analysis of experimentally verified cleavage sites. Protein Sci 13: 2819–2824. 15340161 10.1110/ps.04682504 PMC2286551

[18] Nakai K (2000) Protein sorting signals and prediction of subcellular localization. Adv Protein Chem 54: 277–344. 10829231 10.1016/s0065-3233(00)54009-1

[19] Kaleem A, Hoessli DC, Haq IU, Walker-Nasir E, Butt A, et al (2011) CREB in long-term potentiation in hippocampus: role of post-translational modifications-studies In silico. J Cell Biochem 112: 138–146. 21053365 10.1002/jcb.22909

[20] Weaver TE (1998) Synthesis, processing and secretion of surfactant proteins B and C. Biochim Biophys Acta. 1408: 173–179. 10.1016/s0925-4439(98)00066-0 9813310

[21] Guttentag S, Robinson L, Zhang P, Brasch F, Bühling F, et al (2003) Cysteine protease activity is required for surfactant protein B processing and lamellar body genesis. Am J Respir Cell Mol Biol 28: 69–79. 12495934 10.1165/rcmb.2002-0111OC

[22] Kaznessis YN, Kim S, Larson RG (2002) Specific mode of interaction between components of model pulmonary surfactants using computer simulations. J Mol Biol 322: 569–582. 12225750 10.1016/s0022-2836(02)00774-x

[23] Kandasamy SK, Larson RG (2005) Molecular dynamics study of the lung surfactant peptide SP-B1–25 with DPPC monolayers: insights into interactions and peptide position and orientation. Biophys J 88: 1577–1592. 15738465 10.1529/biophysj.104.038430 PMC1305215

[24] Duncan SL, Larson RG (2010) Folding of lipid monolayers containing lung surfactant proteins SP-B(1–25) and SP-C studied via coarse-grained molecular dynamics simulations. Biochim Biophys Acta 1798: 1632–1650. 20435014 10.1016/j.bbamem.2010.04.006

[25] Baoukina S, Tieleman DP (2011) Lung surfactant protein SP-B promotes formation of bilayer reservoirs from monolayer and lipid transfer between the interface and subphase. Biophys J 100: 1678–1687. 21463581 10.1016/j.bpj.2011.02.019 PMC3072669

[26] Krieger E, Koraimann G, Vriend G (2002) Increasing the precision of comparative models with YASARA NOVA–a self-parameterizing force field. Proteins 47: 393–402. 11948792 10.1002/prot.10104

[27] Krieger E, Joo K, Lee J, Lee J, Raman S, et al (2009) Improving physical realism, stereochemistry, and side-chain accuracy in homology modeling: Four approaches that performed well in CASP8. Proteins 77 Suppl 9114–122. 19768677 10.1002/prot.22570 PMC2922016

[28] Zhang Y (2007) Template-based modeling and free modeling by I-TASSER in CASP7. Proteins 69 Suppl 8108–117. 17894355 10.1002/prot.21702

[29] Roy A, Kucukural A, Zhang Y (2010) I-TASSER: a unified platform for automated protein structure and function prediction. Nat Protoc 5: 725–738. 20360767 10.1038/nprot.2010.5 PMC2849174

[30] Vallat BK, Pillardy J, Elber R (2008) A template-finding algorithm and a comprehensive benchmark for homology modeling of proteins. Proteins 72: 910–928. 18300226 10.1002/prot.21976 PMC2907141

[31] Vallat BK, Pillardy J, Majek P, Meller J, Blom T, et al (2009) Building and assessing atomic models of proteins from structural templates: learning and benchmarks. Proteins 76: 930–945. 19326457 10.1002/prot.22401 PMC2719020

[32] Kim DE, Chivian D, Baker D (2004) Protein structure prediction and analysis using the Robetta server. Nucleic Acids Res 32: W526–W531. 15215442 10.1093/nar/gkh468 PMC441606

[33] Laskowski RA, MacArthur DS, Moss DS, Thornton JM (1993) PROCHECK: a program to check the stereochemical quality of protein structures. J Appl Cryst 26: 283–291.

[34] Sippl MJ (1993) Recognition of errors in three-dimensional structures of proteins. Proteins 17: 355–362. 8108378 10.1002/prot.340170404

[35] Castrignanò T, De Meo PD, Cozzetto D, Talamo IG, Tramontano A (2006) The PMDB Protein Model Database. Nucleic Acids Res 34: D306–309. 16381873 10.1093/nar/gkj105 PMC1347467

[36] Krieger E, Darden T, Nabuurs SB, Finkelstein A, Vriend G (2004) Making optimal use of empirical energy functions: force-field parameterization in crystal space. Proteins 57: 678–683. 15390263 10.1002/prot.20251

[37] Krieger E, Nielsen JE, Spronk CA, Vriend G (2006) Fast empirical pKa prediction by Ewald summation. J Mol Graph Model 25: 481–486. 16644253 10.1016/j.jmgm.2006.02.009

[38] Artimo P, Jonnalagedda M, Arnold K, Baratin D, Csardi G, et al (2012) ExPASy: SIB bioinformatics resource portal. Nucleic Acids Res 40: W597–W603. 22661580 10.1093/nar/gks400 PMC3394269

[39] Kiemer L, Bendtsen JD, Blom N (2005) NetAcet: prediction of N-terminal acetylation sites. Bioinformatics 21: 1269–1270. 15539450 10.1093/bioinformatics/bti130

[40] Blom N, Sicheritz-Ponten T, Gupta R, Gammeltoft S, Brunak S (2004) Prediction of post-translational glycosylation and phosphorylation of proteins from the amino acid sequence. Proteomics 4: 1633–1649. 15174133 10.1002/pmic.200300771

[41] Julenius K, Molgaard A, Gupta R, Brunak S (2005) Prediction, conservation analysis, and structural characterization of mammalian mucin-type O-glycosylation sites. Glycobiology 15: 153–164. 15385431 10.1093/glycob/cwh151

[42] Gupta R, Brunak S (2002) Prediction of glycosylation across the human proteome and the correlation to protein function. Pac Symp Biocomput: 310–322. 11928486

[43] Blom N, Gammeltoft S, Brunak S (1999) Sequence and structure-based prediction of eukaryotic protein phosphorylation sites. J Mol Biol 294: 1351–1362. 10600390 10.1006/jmbi.1999.3310

[44] Ren J, Wen L, Gao X, Jin C, Xue Y, et al (2008) CSS-Palm 2.0: an updated software for palmitoylation sites prediction. Protein Eng Des Sel 21: 639–644. 18753194 10.1093/protein/gzn039 PMC2569006

[45] Yu SH, Possmayer F (2003) Lipid compositional analysis of pulmonary surfactant monolayers and monolayer-associated reservoirs. J Lipid Res 44: 621–629. 12562850 10.1194/jlr.M200380-JLR200

[46] Alonso C, Alig T, Yoon J, Bringezu F, Warriner H, et al (2004) More than a monolayer: relating lung surfactant structure and mechanics to composition. Biophys J 87: 4188–4202. 15454404 10.1529/biophysj.104.051201 PMC1304928

[47] van der Spoel D, Lindahl E, Hess B, Groenhof G, Mark AE, et al (2005) GROMACS: fast, flexible, and free. J Comput Chem 26: 1701–1718. 16211538 10.1002/jcc.20291

[48] Hess B, Kutzner C, van der Spoel D, Lindahl E (2008) GROMACS 4: Algorithms for Highly Efficient, Load-Balanced, and Scalable Molecular Simulation. J Chem Theory Comput 4: 435–447. 26620784 10.1021/ct700301q

[49] Oostenbrink C, Villa A, Mark AE, van Gunsteren WF (2004) A biomolecular force field based on the free enthalpy of hydration and solvation: the GROMOS force-field parameter sets 53A5 and 53A6. J Comput Chem 25: 1656–1676. 15264259 10.1002/jcc.20090

[50] Kukol A (2009) Lipid Models for United-Atom Molecular Dynamics Simulations of Proteins. J Chem Theory Comput 5: 615–626. 26610227 10.1021/ct8003468

[51] Smith GR (2002) G43a1 force field modified to contain phosphorylated Ser, Thr and Tyr. GROMACS User Contributions. Available: http://www.gromacs.org/Downloads/User_contributions/Force_fields.

[52] Sommer B, Dingersen T, Gamroth C, Schneider SE, Rubert S, et al (2011) CELLmicrocosmos 2.2 MembraneEditor: A Modular Interactive Shape-Based Software Approach to Solve Heterogeneous Membrane Packing Problems. J Chem Inf Model 51: 1165–1182. 21504163 10.1021/ci1003619

[53] Nosé‚ S (1984) A molecular dynamics method for simulations in the canonical ensemble. Mol Phys 52: 255–268.

[54] Hoover WG (1985) Canonical dynamics: Equilibrium phase-space distributions. Phys Rev A 31: 1695–1697. 10.1103/physreva.31.1695 9895674

[55] Parrinello M, Rahman A (1981) Polymorphic transitions in single crystals: A new molecular dynamics method. J Appl Phys 52: 7182–7190.

[56] Nosé‚ S, Klein ML (1983) Constant pressure molecular dynamics for molecular systems. Mol Phys 50: 1055–1076.

[57] Hess B, Bekker H, Berendsen HJC, Fraaije JGEM (1997) LINCS: A linear constraint solver for molecular simulations. J Comput Chem 18: 1463–1472.

[58] Hess B (2008) P-LINCS: A parallel linear constraint solver for molecular simulation. J Chem Theory Comput 4: 116–122. 26619985 10.1021/ct700200b

[59] Darden T, York D, Pedersen L (1993) Particle mesh Ewald: An N*log(N) method for Ewald sums in large systems. J Chem Phys 98: 10089–10092.

[60] Essmann U, Perera L, Berkowitz ML, Darden T, Lee H, et al (1995) A smooth particle mesh Ewald method. J Chem Phys 103: 8577–8593.

[61] Humphrey W, Dalke A, Schulten K (1996) VMD: visual molecular dynamics. J Mol Graph 14: 33–38. 8744570 10.1016/0263-7855(96)00018-5

[62] Whitsett JA, Hull WM, Ohning B, Ross G, Weaver TE (1986) Immunologic identification of a pulmonary surfactant-associated protein of molecular weight = 6000 daltons. Pediatr Res 20: 744–749. 3526265 10.1203/00006450-198608000-00009

[63] Yu SH, Chung W, Olafson RW, Harding PG, Possmayer F (1987) Characterization of the small hydrophobic proteins associated with pulmonary surfactant. Biochim Biophys Acta 921: 437–448. 3663690 10.1016/0005-2760(87)90070-1

[64] King RJ, Klass DJ, Gikas EG, Clements JA (1973) Isolation of apoproteins from canine surface active material. Am J Physiol 224: 788–795. 4633419 10.1152/ajplegacy.1973.224.4.788

[65] van Iwaarden F, Welmers B, Verhoef J, Haagsman HP, van Golde LM (1990) Pulmonary surfactant protein A enhances the host-defense mechanism of rat alveolar macrophages. Am J Respir Cell Mol Biol 2: 91–98. 2306370 10.1165/ajrcmb/2.1.91

[66] Mittal RA, Hammel M, Schwarz J, Heschl KM, Bretschneider N, et al (2012) SFTA2-A Novel Secretory Peptide Highly Expressed in the Lung-Is Modulated by Lipopolysaccharide but Not Hyperoxia. PLoS ONE 7: e40011. 22768197 10.1371/journal.pone.0040011 PMC3386909

[67] Gustafsson M, Palmblad M, Curstedt T, Johansson J, Schürch S (2000) Palmitoylation of a pulmonary surfactant protein C analogue affects the surface associated lipid reservoir and film stability. Biochim Biophys Acta 1466: 169–178. 10825440 10.1016/s0005-2736(00)00198-x

[68] Glasser SW, Korfhagen TR, Weaver TE, Clark JC, Pilot-Matias T, et al (1988) cDNA, deduced polypeptide structure and chromosomal assignment of human pulmonary surfactant proteolipid, SPL(pVal). J Biol Chem 263: 9–12. 3335510

[69] Voorhout WF, Veenendaal T, Haagsman HP, Weaver TE, Whitsett JA, et al (1992) Intracellular processing of pulmonary surfactant protein B in an endosomal/lysosomal compartment. Am J Physiol 263: L479–486. 1415726 10.1152/ajplung.1992.263.4.L479

[70] Beck DC, Ikegami M, Na C-L, Zaltash S, Johansson J, et al (2000) The Role of Homodimers in Surfactant Protein B Function in Vivo. J Biol Chem 275: 3365–3370. 10652327 10.1074/jbc.275.5.3365

[71] ten Brinke A, Vaandrager AB, Haagsman HP, Ridder ANJA, van Golde LMG, et al (2002) Structural requirements for palmitoylation of surfactant protein C precursor. Biochem J 361: 663–671. 11802797 10.1042/0264-6021:3610663 PMC1222350

[72] Vorbroker DK, Voorhout WF, Weaver TE, Whitsett JA (1995) Posttranslational processing of surfactant protein C in rat type II cells. Am J Physiol 269: L727–733. 8572234 10.1152/ajplung.1995.269.6.L727

[73] Khoor A, Stahlman MT, Gray ME, Whitsett JA (1994) Temporal-spatial distribution of SP-B and SP-C proteins and mRNAs in developing respiratory epithelium of human lung. J Histochem Cytochem 42: 1187–1199. 8064126 10.1177/42.9.8064126

[74] Bernhard W, Haagsman HP, Tschernig T, Poets CF, Postle AD, et al (1997) Conductive airway surfactant: surface-tension function, biochemical composition, and possible alveolar origin. Am J Respir Cell Mol Biol 17: 41–50. 9224208 10.1165/ajrcmb.17.1.2594

[75] Weston WM, LeClair EE, Trzyna W, McHugh KM, Nugent P, et al (1999) Differential display identification of plunc, a novel gene expressed in embryonic palate, nasal epithelium, and adult lung. J Biol Chem 274: 13698–13703. 10224143 10.1074/jbc.274.19.13698

[76] LeClair EE, Nguyen L, Bingle L, MacGowan A, Singleton V, et al (2001) Genomic organization of the mouse plunc gene and expression in the developing airways and thymus. Biochem Biophys Res Commun 284: 792–797. 11396972 10.1006/bbrc.2001.5024

[77] Bartlett J, Gakhar L, Penterman J, Singh P, Mallampalli RK, et al (2011) PLUNC: a multifunctional surfactant of the airways. Biochem Soc Trans 39: 1012–1016. 21787339 10.1042/BST0391012 PMC3572202

[78] Schicht M, Posa A, Paulsen F, Bräuer L (2010) The ocular surfactant system and its relevance in the dry eye. Klin Monbl Augenheilkd 227: 864–70. 21077020 10.1055/s-0029-1245609

[79] Bräuer L, Paulsen F (2008) Tear film and ocular surface surfactants. J Epithelial BiolPharmacol 1: 62–67.

[80] Paananen R, Glumoff V, Sormunen R, Voorhout W, Hallman M (2001) Expression and localization of lung surfactant protein B in Eustachian tube epithelium. Am J Physiol Lung Cell Mol Physiol 280: L214–220. 11158999 10.1152/ajplung.2001.280.2.L214

[81] Kankavi O (2003) Immunodetection of surfactant proteins in human organ of Corti, Eustachian tube and kidney. Acta Biochim Pol 50: 1057–1064. 14739994

[82] Annalaura Mancia, Spyropoulos DD, McFee WE, Newton DA, Baatz JE (2012) Cryopreservation and in vitro culture of primary cell types from lung tissue of a stranded pygmy sperm whale (Kogia breviceps). Comp Biochem Physiol C Toxicol Pharmacol 155: 136–142. 21501697 10.1016/j.cbpc.2011.04.002 PMC3158290

